# From development to disease: the neuronal role of hnRNPs through the lens of *Drosophila*

**DOI:** 10.3389/fcell.2026.1819111

**Published:** 2026-05-28

**Authors:** Chiara Stefanelli, Davide Colaianni, Cristiano De Pittà, Gabriella M. Mazzotta

**Affiliations:** Department of Biology, University of Padova, Padova, Italy

**Keywords:** *Drosophila*, heterogeneous nuclear ribonucleoproteins, neurodegeneration, neurodevelopement, RNA metabolism

## Abstract

Heterogeneous nuclear ribonucleoproteins (hnRNPs) are a large family of RNA-binding proteins (RBPs) involved in multiple aspects of nucleic acid metabolism, ranging from DNA maintenance to RNA splicing and translation. As a consequence, hnRNPs play a pivotal role during development and in cellular homeostasis, and their dysregulation has been linked to a wide range of diseases, in particular cancer and neurodegenerative disorders. Owing to the high evolutionary conservation of their domains and functions, the fruit fly *Drosophila melanogaster* represents a useful model for the study and characterisation of different families of RBPs. To date, 14 hnRNPs homologous to mammalian proteins have been identified in fruit flies. Here, we review the main *Drosophila* hnRNPs and their parallels with mammalian counterparts, focusing on their functional roles in neuronal development and neurophysiological processes and, in particular, neurodegenerative diseases. Importantly, we discuss the current use of *Drosophila* models in the study of hnRNP-related neurodegenerative conditions and highlight the still largely unexplored potential of this organism in unveiling their underlying molecular mechanisms.

## 1 Introduction

Heterogeneous nuclear ribonucleoproteins (hnRNPs) comprise a large and diverse family of RNA-binding proteins (RBPs). Initially identified through their association with heterogeneous nuclear RNA (hnRNA) in ribonucleoprotein complexes (Beyer et al., 1977), hnRNPs were later shown to play far broader and more fundamental roles in nucleic acid biology. Notably, several hnRNP family members were first isolated and functionally characterised through studies of the *Drosophila* germline and early embryo, highlighting their central role in developmental RNA regulation from the earliest stages (Matunis E. L. et al., 1992; Matunis M. J. et al., 1992). hnRNPs are now recognised as multifunctional regulators involved in numerous aspects of DNA and RNA metabolism, with a significant impact on development and disease (Low et al., 2021).

### 1.1 hnRNPs in RNA metabolism

**FIGURE 1:**
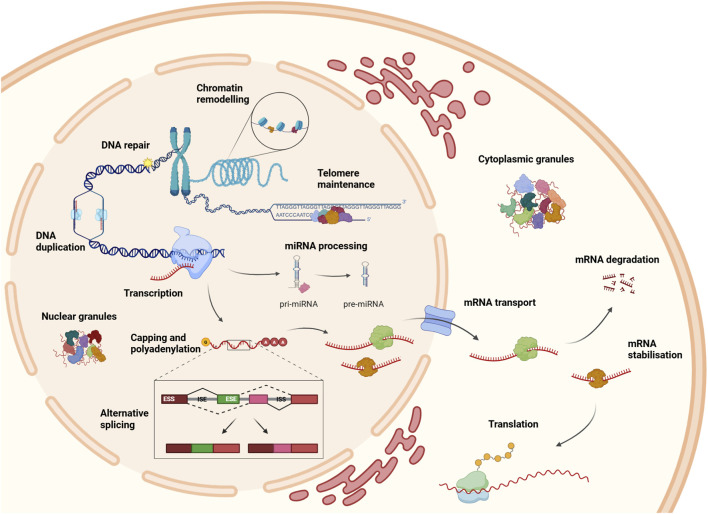
Summary of hnRNPs main functions across DNA and RNA metabolism. Created with BioRender.com.

hnRNPs are integral to all phases of nucleic acid metabolism. They function in DNA replication, repair, and telomere maintenance, as well as chromatin remodelling. They play key roles in RNA transcription, processing, alternative splicing regulation, and microRNA (miRNA) maturation. Additionally, hnRNPs facilitate RNA nucleocytoplasmic transport and modulate both mRNA translation and stability (Dreyfuss et al., 2002; Lo Piccolo et al., 2014) (Figure 1). hnRNPs are also important components of intracellular RNA granules, through which they participate in the stress response (Ripin and Parker, 2023; Chujo et al., 2016). In the following subsections, we focus on the most prominent functions exerted by hnRNPs at the RNA level.

#### 1.1.1 Alternative splicing

One of the most studied molecular functions of hnRNPs is their role in alternative splicing, the cellular process that produces different forms of mature mRNAs from a single pre-mRNA, facilitating interactions between cis-regulatory elements and trans-acting factors that recruit the spliceosome complex (Wang et al., 2015) (Figure 1). hnRNPs function in coordination with serine/arginine-rich (SR) proteins: hnRNPs typically act as splicing repressors by binding exonic and intronic splicing silencers (ESSs and ISSs), while SR proteins promote splicing by binding splicing enhancers (ESEs and ISEs) (Busch and Hertel, 2012). Binding of hnRNPs and SR proteins to their respective sites influences the formation of the pre-E complex, the first step of spliceosome assembly, by modulating the recruitment of small nuclear ribonucleoproteins (snRNPs) to the pre-mRNA, determining whether an exon is skipped or included in the final mRNA product (Busch and Hertel, 2012; Tao et al., 2024). The effects of hnRNPs and SR proteins are highly context-dependent and can vary depending on their binding position relative to splice sites (Erkelenz et al., 2013). In addition, these proteins engage in both cooperative and antagonistic interactions, not only within their own families but also between hnRNPs and SR proteins, further increasing the complexity of splicing regulation (Huelga et al., 2012; Bradley et al., 2015; Brooks et al., 2015). Furthermore, certain hnRNPs or subfamilies exhibit unique splicing mechanisms. For example, hnRNP A1 can bind a high-affinity site and cooperatively recruit additional hnRNP A1 molecules, spreading along the pre-mRNA and displacing the other proteins. This effectively masks splice sites and represses splicing (Okunola and Krainer, 2009). Similarly, hnRNP A/B proteins and hnRNP F can bind high-affinity sites within the same or different introns and form dimers that loop out RNA regions. This looping brings distant splice sites closer together, helping define intron boundaries and regulate exon inclusion or skipping. Through these mechanisms, hnRNPs influence splicing by shaping pre-mRNA structure (Martinez-Contreras et al., 2006; Fisette et al., 2010).

#### 1.1.2 mRNA nucleocytoplasmic transport

The majority of hnRNPs primarily reside in the nucleus due to their nuclear localisation domain, but some can shuttle between the nucleus and the cytoplasm (Dreyfuss et al., 1993) (Figure 1). This movement is usually coupled with mRNA transport, allowing the temporal and spatial control of gene expression. In the nervous system, such regulation is crucial for axon maintenance and growth, and formation of dendritic synapses (Rangaraju et al., 2017; Bramham and Wells, 2007). hnRNPs act as trans-acting factors that recognise specific cis-acting elements within the mRNA untranslated regions, such as the RNA trafficking sequence (RTS), and assemble into messenger ribonucleoprotein (mRNP) complexes that exit the nucleus via nuclear pore complexes (NPCs) (Ainger et al., 1997; Raju et al., 2008; Cullen, 2000). Some hnRNPs with nuclear retention signals, such as hnRNP C and hnRNP U, mediate mRNA shuttling but dissociate before export (Piñol-Roma and Dreyfuss, 1992; Mili et al., 2001), whereas others, including hnRNP A1, hnRNP F, and hnRNP K, remain attached to mRNA during export and can shuttle back into the nucleus (Piñol-Roma and Dreyfuss, 1992; Mili et al., 2001; Lee et al., 2018). After export, the composition of mRNP is remodelled to initiate translation, a process also regulated by hnRNPs (Torvund-Jensen et al., 2014). For instance, hnRNP A1, hnRNP Q1, and hnRNP F bind G-quadruplex mRNA structures (RG4) and modulate translation either positively or negatively (Dabral et al., 2020; Williams et al., 2016; Herviou et al., 2020). Notably, hnRNP F preferentially binds the unfolded form of RG4 after the unwinding by RNA helicase DHX36, thereby maintaining RNA in a linear conformation (Herviou et al., 2020).

#### 1.1.3 Intracellular granules formation

hnRNPs are also important components of nuclear and cytoplasmic granules, membrane-less organelles formed via a process known as liquid-liquid phase separation. Physiological granules in the nucleus are often scaffolded by long non-coding RNAs (lncRNAs), termed architectural RNAs (arcRNAs) (Chujo et al., 2016) (Figure 1). These granules exert important cellular functions as they can act as hubs for interactions between RBPs and other kinds of proteins, but some of them are also implicated in the stress response (Singh, 2022; Chujo et al., 2016). To date, only six arcRNA have been reported in the literature across studied organisms, with NEAT1 and SatIII in humans and hsrω in *D*. *melanogaster* being associated with different hnRNPs in distinct nuclear granules, specifically paraspeckles, nuclear stress bodies, and omega speckles (Chujo et al., 2016; Grzejda et al., 2022). Intracellular granules, in both the nucleus and the cytoplasm, can also form in response to stress (*e*.*g*., oxidative or heat stress), in order to preserve inactive mRNAs. Granule formation can be mediated by the intrinsically disordered regions (IDRs) of hnRNPs; however, mutations within these proteins can promote the formation of insoluble, toxic aggregates in neuronal cells (Lin et al., 2015; Jiang et al., 2023). Similar pathogenic aggregates may also originate from mRNAs harbouring expanded repeat sequences, which sequester key regulatory factors, including hnRNPs, thereby impairing their normal function (Jiang et al., 2023).

### 1.2 hnRNPs structure and domain architecture

**FIGURE 2:**
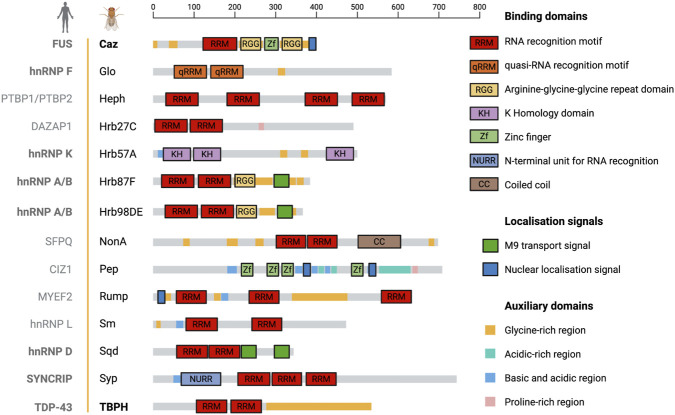
*Drosophila* hnRNPs structure. Representation of *Drosophila* hnRNPs domain composition according to UniProt records (Caz, Q27294; Glo, Q9VGH5; Heph, Q7KES3; Hrb27C, P48809; Hrb57A, A1ZBW0; Hrb87F, P48810; Hrb98DE, P07909; NonA, Q04047; Pep, P41073; Rump, Q9VHC7; Sm, Q7JMZ7; Sqd, Q08473; Syp, A0A0B4KHI4; TBPH, O97468) and available literature (Caz, Bogaert et al., 2018; Glo, Warden et al., 2023; Heph, Dansereau et al., 2002; Hrb98DE, Ji and Tulin, 2009; Pep, Amero et al., 1991; Rump, Johnston et al., 2017; Sqd, Norvell et al., 1999; Syp, Hobor et al., 2018; TBPH, Kushimura et al.,2018). *Drosophila* hnRNPs are indicated in black, while their human homologue(s), defined accordingly to FlyBase scores, are indicated in grey. The bold text highlights well-established human hnRNPs whose mutations are linked to neurodegenerative and neurodevelopmental disorders (Gillentine et al., 2021; Gillentine, 2025). (Hrb87F and Hrb98DE are widely recognised as homologues of the subfamily hnRNP A/B comprising hnRNP A1 and hnRNP A2/B1). Created with BioRender.com.

hnRNPs exhibit a shared structural organisation that is broadly conserved across species, highlighting their essential functions in nucleic acid metabolism. Figure 2 provides an overview of the architecture of the major *Drosophila* hnRNPs, highlighting the distribution and organisation of their domains. The human homologues are also reported. All hnRNPs possess one or more RNA-binding domains (RBDs), with the RNA recognition motif (RRM) being the most prevalent one. This domain is composed of 90 amino acids organised in a *β*_1_ *α*_1_ *β*_2_ *β*_3_ *α*_2_ *β*_4_ topology. It primarily binds single-stranded RNA (ssRNA) but is also capable of recognising single-stranded DNA (ssDNA) and can participate in protein-protein interactions (Maris et al., 2005). These interactions rely on three aromatic residues in two highly conserved sequences (RNP1 and RNP2) situated in *β*3 and *β*1, respectively. When these aromatic residues are absent, the domain is referred as quasi-RRM (qRRM), in which the binding of the RNA is mediated primarily by the protein loops rather than the canonical β-sheet interactions (Cléry et al., 2008). Another key RNA-binding domain is the K Homology (KH) domain, 70 amino acids that, in eukaryotes, adopt a *β*_1_ *α*_1_ *α*_2_ *β*_2_ *β*_3_ *α*_3_ topology. Recognition of ssRNA and ssDNA occurs via a binding cleft capable of accommodating four nucleotides. This cleft is formed by the *α*1 and *α*2 helices, the GXXG loop connecting them, the adjacent *β*2-sheet, and a variable loop at the C-terminal end (Valverde et al., 2008). hnRNPs also contain the arginine-glycine-glycine (RGG) repeat domain, which, although often classified as auxiliary, actively contributes to binding (Chowdhury and Jin, 2023). This low-complexity flexible motif consists of Arg-Gly-Gly tripeptide repeat, typically flanked by hydrophobic and aromatic residues. This versatile domain is often associated with one or more of the structured domains described above, enabling more flexible interaction with nucleic acids and proteins (Chowdhury and Jin, 2023). Other auxiliary domains, typically rich in proline, glycine, or acidic amino acids, can be present and are usually found in IDRs that contribute to a dynamic structure and a broad set of interactions (Dreyfuss et al., 1993; Biamonti and Riva, 1994; Calabretta and Richard, 2015; Geuens et al., 2016). Moreover, localisation or import/export sequences can also be present, such as nuclear localisation signals (NLS) or the acidic M9 transport signal, a proline-tyrosine nuclear localisation signal (PY-NLS) present in the hnRNP A/B subfamily, which comprises hnRNP A1 and hnRNP A2/B1 (Görlich, 1997; Izaurralde et al., 1997; Lee et al., 2006). Consistent with the high degree of structural conservation, the hnRNP repertoire of *Drosophila melanogaster* displays a specific combination of conserved binding domains, auxiliary domains, and localisation signals.

## 2 hnRNPs in humans and flies

Mammalian hnRNPs are grouped into 20 subfamilies based on their domain composition and structure. These subfamilies, named from A to U, exhibit considerable diversity, with each subfamily encompassing multiple paralogue genes, and individual genes often producing several isoforms (Piñol-Roma et al., 1988). *Drosophila melanogaster* has proven to be a valuable model to elucidate both ubiquitous and tissue-specific functions of RBPs (Gamberi et al., 2006; Olesnicky and Wright, 2018; Takeiwa et al., 2024). In fruit flies, 14 hnRNPs are widely considered homologous to their mammalian counterparts (Lo Piccolo et al., 2014). In a recent computational analysis, other *Drosophila* proteins have been suggested as potential orthologues based on domain similarity, although most of them still lack a proper characterisation (Nishanth and Jha, 2025). hnRNPs exhibit high structural similarity between *D*. *melanogaster* and humans. For instance, *Drosophila* Heph and TBPH show striking domain conservation with their human orthologues, PTBP1/PTBP2 and TDP-43, respectively, underscoring the deep evolutionary conservation of their functional mechanisms (Dansereau et al., 2002; Ayala et al., 2005). Following the classification by Lo Piccolo et al. (2014), we identified the human orthologues for each *Drosophila* hnRNP using FlyBase (Öztürk-Çolak et al., 2024), that integrates current literature with multiple external genomic projects to ensure high-confidence correspondence between species. In this review, we specifically address the roles of hnRNPs in neurodevelopment and neurodegeneration, highlighting both existing applications and the largely unexplored potential of *Drosophila melanogaster* in investigating hnRNP-related neurodegenerative disorders and their molecular bases.

### 2.1 hnRNPs in the developing nervous system

**FIGURE 3:**
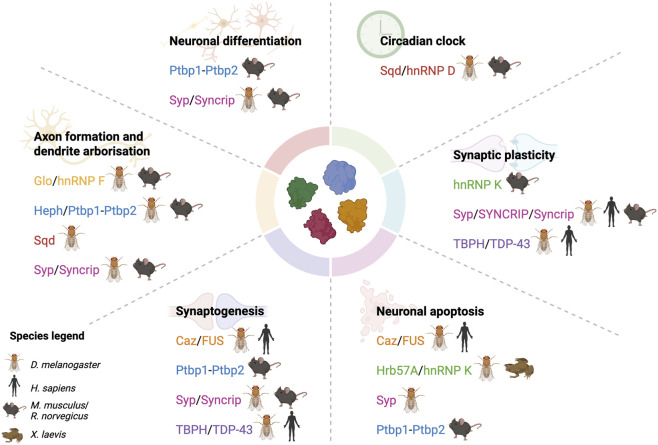
Overview of hnRNPs involvement in neuronal development and physiology. Representation of the key neuronal functions mentioned in this review with parallelism between *Drosophila* hnRNPs and their homologues in other species (*Drosophila melanogaster, Homo sapiens, Mus musculus, Rattus norvegicus, Xenopus laevis*), written in the same colour: Caz and FUS (orange); Sqd and hnRNP D (red); Glo and hnRNP F (yellow); Hrb57A and hnRNP K (green); Heph, PTBP1, PTBP2, Ptbp1, and Ptbp2 (blue); Syp, SYNCRIP, and Syncrip (pink), TBPH and TDP-43 (purple). Created with BioRender.com.

hnRNPs are key regulators of development and cell differentiation (Geuens et al., 2016), as evidenced by studies in animal models ranging from fruit flies to mice, where loss of function of specific hnRNPs leads to early-stage lethality (Layalle et al., 2005; Möröy and Heyd, 2007; Liu et al., 2017) (Figure 3; Table 1). Accordingly, recent studies have further established a fundamental role for hnRNPs in stem cell self-renewal and differentiation (Xie et al., 2021). In humans, defects in their function have been linked to tumour progression (Carpenter et al., 2006) as well as a range of developmental disorders, particular affecting muscle and neuronal tissues (Li et al., 2023; Gillentine, 2025), often reflecting their tissue-specific patterns. For example, during development, both mammalian PTBP1/PTBP2 and *Drosophila* Heph are expressed in neural and myogenic tissues (Liu et al., 2023; Boutz et al., 2007). During muscle differentiation, murine Ptbp1/Ptbp2 are post-transcriptionally regulated by the conserved microRNAs miR-133 and miR-1/206, which are also predicted to target heph and human PTBP1/PTBP2 mRNAs (Boutz et al., 2007). Murine Ptbp1 and Ptbp2 exhibit almost mutually exclusive expression patterns during CNS development. Together, they regulate axon formation, synaptogenesis, and neuronal apoptosis, primarily by acting as repressors of neuron-specific alternative splicing (Liu et al., 2023; Vuong et al., 2016) (Figure 3; Table 1). It should be noted, however, that their regulatory activity usually depends on both their binding position on the pre-mRNA and the sequence context (Hamid and Makeyev, 2017). *Ptbp2* knock-out in mice results in neuronal defects leading to progressive degeneration of the cerebral cortex and postnatal lethality (Vuong et al., 2016); similarly, loss or reduced expression of *heph* in *Drosophila* causes impaired CNS development (Wesley et al., 2011) and significant defects in dendrite arborisation (Aboukilila et al., 2020) (Figure 3; Table 1), reflecting its role as a negative regulator of Notch and Oskar, respectively.

**TABLE 1:** Overview of *Drosophila* hnRNPs and their neuronal functions**.** Summary of hnRNPs in *Drosophila*, along with the corresponding human orthologues, with main roles in specific neuronal processes. Key references are provided for each protein to support the reported functions.

*Drosophila* protein	Human orthologue	Neuronal process	Key references
Caz	FUS	Neuronal apoptosis, synaptogenesis	Ling (2018) Wang et al. (2011) Xia et al. (2012)
Hrb57A	hnRNP K	Neuronal apoptosis, synaptic plasticity	Folci et al. (2014) Mallik and Lakhotia (2009) Wang et al. (2020)
Syp	SYNCRIP	Axon formation and dendrite arborisation, neuronal apoptosis, neuronal differentiation, synaptogenesis, synaptic plasticity	Bannai et al. (2004) Duning et al. (2008) Guan et al. (2024) Pahl et al. (2019) Ren et al. (2017) Titlow et al. (2020) Wu et al. (2025)
Heph	PTBP1/PTBP2	Axon formation and dendrite arborisation, neuronal apoptosis, neuronal differentiation, synaptogenesis	Aboukilila et al. (2020) Liu et al. (2023) Wesley et al. (2011)
Glo	hnRNP F	Axon formation and dendrite arborization	Brechbiel and Gavis (2008) Lee et al. (2018)
Sqd	hnRNP D	Axon formation and dendrite arborisation, circadian clock	Damulewicz et al. (2025) Kim et al. (2015) Olesnicky et al. (2014) Woo et al. (2010)
TBPH	TDP-43	Synaptogenesis, synaptic plasticity	Feiguin et al. (2009) Fiesel et al. (2011) Ling (2018) Wang et al. (2011)

Other key neurodevelopmental factors in *Drosophila*, like CREB-binding protein (CBP) (Mitrousis et al., 2015), also known as Nejire, and *Drosophila* Inhibitor of Apoptosis Protein 1 (DIAP1) (Pinto-Teixeira et al., 2016), a member of the Inhibitor of Apoptosis Protein (IAP) family (Robertson et al., 2000), have been shown to physically interact with Hrb57A within omega speckles (Mallik and Lakhotia, 2009; Mallik and Lakhotia, 2010). Downregulation of *hsrω*, the arcRNA of omega speckles, causes these granules to disassemble, redistributing the associated hnRNPs into the nucleoplasm; this increases Hrb57A-mediated stabilisation of DIAP1, which inhibits caspase activation and suppresses apoptosis in eye and imaginal discs (Mallik and Lakhotia, 2009) (Figure 3; Table 1). This redistribution also reduces CBP in the nuclear granules, leading to its accumulation with Hrb57A in the cytoplasmic granules (Mallik and Lakhotia, 2010). The Hrb57A homologue, hnRNP K, is essential for neural development also in *Xenopus laevis*, where reduced phosphorylation at serine 257 impairs its function and consequently attenuates the axon outgrowth (Wang et al., 2020) (Figure 3; Table 1). In mice, *hnRNPK* haploinsufficiency leads to developmental defects, including neonatal lethality, while mutations in *HNRNPK* in humans result in intellectual disabilities (Wang et al., 2020), further underscoring its role in neurodevelopment. In addition, hnRNP K has been identified as a critical regulator of synaptic transmission and plasticity in rat hippocampal neurons, highlighting its importance in neuronal physiology (Folci et al., 2014; Leal et al., 2017) (Figure 3; Table 1).

The role of hnRNPs in neuronal physiology extend to complex processes such as the regulation of circadian rhythms. In *Drosophila*, *sqd* mutants display impaired circadian locomotor activity (Damulewicz et al., 2025). This phenotype is accompanied by disorganisation of projection from PDF-positive clock neurons (l-LNv and s-LNv), suggesting a structural basis for the behavioural defects (Damulewicz et al., 2025). Such alteration may stem from established role of Sqd in dendrite morphogenesis, as previously demonstrated in sensory neurons (Olesnicky et al., 2014; Olesnicky et al., 2018). Consistent with a conserved function, the Sqd homologue hnRNP D has been identified as a fine modulator of circadian rhythmicity in mammals, where it promotes the destabilisation of both mouse Cryptochrome 1 (mCry1) (Woo et al., 2010) and Period3 (mPer3) (Kim et al., 2015) mRNAs (Figure 3; Table 1).

Other hnRNPs with pleiotropic effects on neuronal development and physiology, conserved across *Drosophila* and humans, are Syp/SYNCRIP, Caz/FUS, and TBPH/TDP-43. Syp, together with IGF-II mRNA-binding protein (Imp), regulates temporal fate specification in *Drosophila* neural lineages through opposing temporal gradients, with increasing Syp promoting late identities (Liu et al., 2015; Ren et al., 2017; Pahl et al., 2019) (Figure 3; Table 1). Mechanistically, in the mushroom body (MB) lineages, Syp and Imp control the translation of the temporal transcription factor Chinmo (Liu et al., 2015). Elevated Syp levels in both MB and non-MB neuroblasts further promote the accumulation of Prospero, a key regulator of the transition from proliferation to differentiation (Yang et al., 2017; Samuels et al., 2020). Supporting this role, the *in vivo* RNA interactomes of Syp and Imp across early and late stages of larval brain development have recently been characterised, highlighting the breadth of their post-transcriptional regulatory networks (Lee et al., 2025). Indeed, Imp and Syp targets are involved in a wide range of biological and molecular processes, including the regulation of NSC growth and quiescence, energy metabolism, intracellular signalling, hormone responses, and tumorigenic potential (Lee et al., 2025). The opposing expression patterns of Syp and Imp are also critical in immature motoneurons (iMNs), where they regulate the translation of multiple transcription factors to control cell fate decisions, with Imp promoting survival and Syp favouring programmed cell death (Guan et al., 2022; Guan et al., 2024) (Figure 3; Table 1). Beyond developmental contexts, Syp functions at the larval neuromuscular junction (NMJ), where it is postsynaptically expressed and acts as a negative regulator of synapse growth (McDermott et al., 2014; Titlow et al., 2020). It also contributes to activity-dependent synaptic plasticity and proper synaptic transmission, by maintaining the appropriate pool of glutamatergic vesicles (Halstead et al., 2014; Titlow et al., 2020) (Figure 3; Table 1). In mammals, the Syp homologues mouse Syncrip and human SYNCRIP perform analogous functions. They regulate the formation of mRNA granule and their dendritic transport, processes essential for synaptic plasticity (Bannai et al., 2004; Duning et al., 2008). Consistent with its *Drosophila* counterpart, murine Syncrip binds a broad set of mRNAs encoding for proteins involved in neurogenesis, neuronal migration, and neurite outgrowth, and contributes to the regulation of temporal transcriptional programs (Khudayberdiev et al., 2021; Wu et al., 2025) (Figure 3; Table 1).

*Drosophila* TBPH and Caz, along with their mammalian counterparts TDP-43 and FUS, are among the most extensively studied hnRNPs, due to their central involvement in neurodegenerative diseases, particularly amyotrophic lateral sclerosis (ALS) (Aksoy et al., 2020; Wang et al., 2011). Beyond their pathological relevance, these proteins play evolutionarily conserved roles in neuronal development, particularly in synapse formation and maturation (Ling, 2018). In *Drosophila*, TBPH is essential for the assembly and organisation of motoneurons terminal branches and synaptic boutons at neuromuscular junctions (Feiguin et al., 2009) (Figure 3; Table 1). Mechanistically, TBPH regulates the translation of futsch mRNA, which encodes a neuron-specific protein required for microtubule organisation at presynaptic terminals (Godena et al., 2011). In humans TDP-43 similarly promotes neurite outgrowth through the post-transcriptional regulation of HDAC6, a cytoskeleton-associated deacetylase (Fiesel et al., 2011) (Figure 3; Table 1). TBPH also controls neuromuscular synapse formation and growth by regulating the expression of the synaptic scaffold protein Disc-large (Dlg), thereby coordinating both pre- and postsynaptic maturation to promote NMJs formation (Strah et al., 2020). Consistently with these roles, *TBPH* loss-of-function models exhibit impaired synaptic transmission and motor defects (Diaper et al., 2013). Conversely, overexpression of *caz* or human *FUS*, as well as *TBPH* or human *TDP-43*, in *Drosophila* motor neurons induces NMJ terminal expansion and increases synaptic bouton number (Wang et al., 2011) (Figure 3; Table 1). Notably, during NMJ terminal expansion, Caz acts downstream of TBPH, indicating a genetic interaction between these hnRNPs (Wang et al., 2011). However, expression of human FUS or *Drosophila* Caz can also disrupt presynaptic architecture and induce motor neuron disorganisation in the larval ventral nerve cord, likely due to apoptosis-induced toxicity (Xia et al., 2012) (Figure 3; Table 1). Similarly, *caz* downregulation leads to motoneurons degeneration and locomotive impairment (Sasayama et al., 2012). In addition to its roles at the NMJ, Caz participates in the EGFR signalling pathway, which is required for cone cells differentiation (Shimamura et al., 2014) and contributes to pre-piRNAs biogenesis in the central nervous system (Wakisaka et al., 2019).

Finally, it is worth noting that additional hnRNP family members also contribute to the nervous system development and physiology. However, current evidence suggests that they often act in distinct neuronal compartments and/or through different biological processes in *Drosophila melanogaster* and humans. For instance, the *Drosophila* hnRNP Glo regulates dendrite growth of larval class IV dendritic arborisation (da) neurons by binding to the translational repressor Nanos (Nos) (Brechbiel and Gavis, 2008) (Figure 3; Table 1). In contrast, its rat homologue hnRNP F promotes axon growth by interacting with the axonal localising motifs of Nrn1 and Hmgb1 mRNAs (Lee et al., 2018) (Figure 3; Table 1).

Taken together, these findings highlight an evolutionarily conserved role for hnRNPs in neuronal development and physiology across *Drosophila*, humans, and other species. Nonetheless, additional functions, and potential deeper cross-species beyond those summarised in Figure 3 and Table 1, are likely to emerge with further investigation.

### 2.2 hnRNPs in neurodegenerative diseases

**FIGURE 4:**
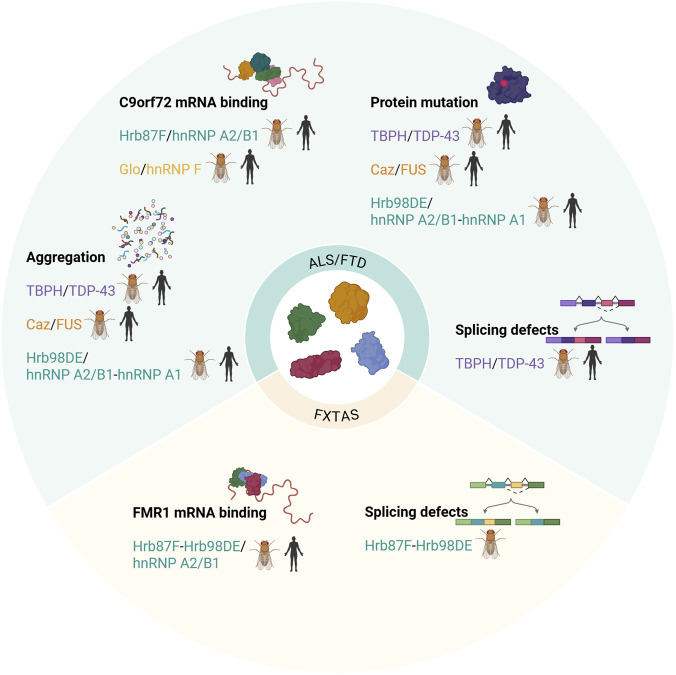
Overview of hnRNPs involvement in ALS/FTD and FXTAS. Representation of ALS/FTD and FXTAS disease mechanisms with parallelism between *Drosophila* and human hnRNPs. Homologues are represented with the same colour: Caz and FUS (orange); Glo and hnRNP F (yellow); Hrb87F, Hrb98DE, hnRNP A1/B2, and hnRNP A1 (green); TBPH and TDP-43 (purple). Created with BioRender.com.

Numerous human hnRNPs have been linked to a range of neurodegenerative diseases and are the subject of several comprehensive reviews (Low et al., 2021; Tilliole et al., 2024; Brandão-Teles et al., 2024; Lourinho et al., 2025). Human hnRNPs associated with neurodegenerative and neurodevelopmental disorders, together with their *Drosophila* counterparts, are highlighted in bold in Figure 2 (Gillentine et al., 2021; Gillentine, 2025). In this section we will focus on *Drosophila* as a model for investigating the pathological roles of hnRNPs in amyotrophic lateral sclerosis (ALS), frontotemporal dementia (FTD), and fragile X-associated tremor/ataxia syndrome (FXTAS) (Figure 4).

#### 2.2.1 Amyotrophic Lateral sclerosis and frontotemporal dementia (ALS/FTD)

ALS and FTD are neurodegenerative disorders characterised primarily by motor dysfunction and cognitive impairment, respectively. However, they share key genetic alterations and often present overlapping clinical features, defining an ALS-FTD spectrum (Abramzon et al., 2020), in which hnRNPs play a central role (Purice and Taylor, 2018). In particular, mutations of *TARDBP* (encoding for TDP-43) and *FUS* are among the most extensively studied (Abramzon et al., 2020; Hegde and Srivastava, 2022). Nevertheless, additional hnRNPs are emerging as contributors to disease pathology (Purice and Taylor, 2018), partially because hnRNPs can cross-regulate each other, such that the dysfunction of a single family member can affect the expression, alternative splicing, or subcellular localisation of others, in both humans and *Drosophila* (Huelga et al., 2012; Brooks et al., 2015; Deshaies et al., 2018; Colaianni et al., 2025; Pinkerton et al., 2024; Hasegawa-Ogawa et al., 2025).

Although the precise mechanisms underlying TDP-43 toxicity are not fully understood, they are thought to act through at least two major pathways. First, the formation of cytoplasmic TDP-43 aggregates, and its consequent nuclear depletion, impairs its nuclear hnRNP function, leading to defects such as alternative polyadenylation (Bryce-Smith et al., 2025; Zeng et al., 2025) and cryptic exon inclusion (Seddighi et al., 2024) (Figure 4). The latter has also been observed in *Drosophila* lacking the TDP-43 homologue TBPH (Donde et al., 2019). Second, the cytoplasmic accumulation of TDP-43 or TBPH can form insoluble toxic granules that sequester essential RNA-binding proteins and other factors (Wood et al., 2021; Ho et al., 2024) (Figure 4). In *Drosophila*, both loss and gain of *TBPH* function result in neuronal defects, particularly affecting synaptic transmission and leading to motor neuron degeneration (Diaper et al., 2013). These phenotypes are mirrored in TDP-43-based models and have extensively reviewed elsewhere, including detailed discussions of TBPH/TDP-43 ALS fly models (Romano et al., 2012). Interestingly, studies in both human and *Drosophila* neuronal cell lines show that TDP-43/TBPH-induced toxicity can be modulated by multiple hnRNPs (Table 2). Specifically, the downregulation of selected hnRNPs can either enhance (*i*.*e*., *sqd* and *nonA*) or suppress (*i*.*e*., *rump*, *heph*, *sm*, *hrb87F*, *hrb27C*, *hrb98DE*, *glo*, and *syp*), to varying degrees, the toxicity observed in *Drosophila TBPH* or *TDP-43* gain-of-function models (Appocher et al., 2017; Koch and Nagoshi, 2025). Conversely, in a *TBPH* loss-of-function *Drosophila* model, depletion of individual hnRNPs significantly affects the impaired climbing phenotype, with the exception of Hrb27C, which results in early lethality (Appocher et al., 2017). Moreover, depletion of *DAZAP1* and *SYNCRIP*, the human homologues of *hrb27C* and *syp* respectively, rescues splicing defects caused by *TDP-43* depletion in the SH-SY5Y neuronal cells, indicating a shared regulation of cellular pathways essential for neuronal function (Appocher et al., 2017). In another *in vitro* system, overexpression of *HNRNPA2B1* was shown to attenuate TDP-43-induced neurotoxicity through direct binding to the protein, suggesting a potential protecting mechanism during disease progression (D’Ambrogio et al., 2009; Suzuki et al., 2015). Consistently, interaction between TDP-43 and the members of the hnRNP A/B subfamily have been implicated in the splicing inhibitory activity of TDP-43 (Buratti et al., 2005). This interaction is evolutionarily conserved in *Drosophila,* where Hrb98DE binds both TBPH and human TDP-43 via conserved protein regions, contributing to splicing modulation (Romano et al., 2014). Moreover, mutations within the IDRs of hnRNPs A2/B1 and A1, as well as equivalent alteration in Hrb98DE, promote protein fibrillization, leading to the formation of insoluble stress granules and subsequent muscle degeneration (Kim et al., 2013) (Figure 4). Among these alterations is the inclusion of a cassette exon, which, in the case of hnRNP A1, may arise from TDP-43 mislocalisation, given that TDP-43 itself regulates *HNRNPA1* pre-mRNA splicing in humans (Deshaies et al., 2018). At the same time, human TDP-43, hnRNP A2/B1, hnRNP A1, and hnRNP L, can regulate the cryptic splicing of *UNC13A*, a major genetic risk factor for ALS/FTD (Brown et al., 2022; Koike et al., 2023).

**TABLE 2:** Genetic modifiers of toxicity in neurodegenerative diseases. Summary of hnRNPs (and hsrω) reported to modify toxicity in ALS/FTD, FXTAS and SCA31 experimental models. Proteins are classified according to their effect as enhancers or suppressors of neurodegenerative phenotypes. OE indicates effects due to overexpression; all the others result from gene downregulation.

Disease	Type of toxicity	Enhancer	Suppressor	References
ALS/FTD	TBPH gain-of-function	Sqd	Glo	Appocher et al. (2017)
		​	Heph	
		​	Hrb27C	
		​	Hrb87F	
		​	Hrb98DE	
		​	Rump	
		​	Sm	
		​	Syp	
	TDP-43 gain-of-function	NonA	​	Koch and Nagoshi (2025)
		​	hnRNP A2/B1^OE^	Suzuki et al. (2015)
		​	hsrω	Chung et al. (2018)
	TBPH loss-of-function	Glo	​	Appocher et al. (2017)
		Heph	​	
		Hrb87F	​	
		Rump	​	
		Sm	​	
		Sqd	​	
		Syp	​	
	TDP-43 loss of function	​	DAZAP1	Appocher et al. (2017)
		​	SYNCRIP	
	C9orf72 gain of function	Hrb87F	hnRNP A2/B1^OE^	Taminato et al. (2023)
FXTAS	CGG repeat expansion	​	hnRNP A2/B1^OE^	Sofola et al. (2007)
		​	Hrb87F^OE^	
		​	Hrb98DE^OE^	
		​	TDP-43^OE^	He et al. (2014)
SCA31	UGGAA repeat expansion	​	FUS^OE^	Ishiguro et al. (2017)
		​	hnRNP A2/B1^OE^	
		TBPH	TDP-43^OE^	
		NonA	​	

TDP-43/TBPH have also been reported to associate with FUS/Caz, although this interaction appears to be RNA-dependent in a context-specific manner. Indeed, RNase A treatment reduces the TBPH/Caz interaction in *Drosophila* brain extracts, whereas no such effect is observed in mammalian cell extracts (Wang et al., 2011; Kim et al., 2010). Notably, both TDP-43 and FUS participate in stress granules assembly in humans: *FUS* overexpression promotes TDP-43 accumulation, while the presence of both proteins is reduced when TDP-43 has impaired RNA-binding capacity (Demongin et al., 2024). Conversely, in ALS/FTD, TDP-43 and FUS more frequently exhibit mutually exclusive aggregation patterns, although partial co-occurrence has also been reported (Portz et al., 2021). In line with this, FUS mislocalisation and occasionally inclusion formation has been observed in TDP-43-mediated ALS patients (Tyzack et al., 2019; Deng et al., 2010) (Figure 4). In *Drosophila*, ALS-associated mutations in FUS/Caz promote inclusion formation, mislocalisation, and redistribution to synaptic terminals in class IV dendritic arborisation (da) neurons, thereby disrupting axonal transport (Machamer et al., 2018) (Figure 4). Importantly, even wild-type *FUS*/*Caz* overexpression alters neuronal physiology, impairing synaptic structure and mitochondria calcium transients (Machamer et al., 2018). Consistently, ALS-associated mutations enhance the interaction between TDP-43 and FUS *in vitro* (Ling et al., 2010). Moreover, co-expression of human wild-type and mutant forms of these proteins in *Drosophila* synergistically exacerbates the neurodegenerative phenotypes, supporting a functional interplay within shared cellular pathways (Lanson et al., 2011) (Figure 4). Interestingly, both earlier and more recent studies have linked Caz/FUS toxicity in *Drosophila* to its nuclear localisation (Jäckel et al., 2015; Moens et al., 2026), a process dependent on transportin activity and modulated by methyltransferases (Jäckel et al., 2015). In fly models expressing human FUS, both dynamic and insoluble aggregates have been observed (Jäckel et al., 2015; Moens et al., 2026), with toxicity associated with interactions between FUS and RNA polymerase II (Polr2A) (Moens et al., 2026). Notably, this genetic interaction may be relevant to FTD pathogenesis, as mislocalisation of POLR2A has also been reported in FUS-positive FTD patients (Moens et al., 2026). Additional evidence for convergence between TDP-43 and FUS pathways comes from studies on long non-coding RNAs (lncRNAs) (Laneve et al., 2021). Both TBPH and Caz physically and functionally interact with the lncRNA hsrω *in vivo* (Lo Piccolo et al., 2018; Lo Piccolo and Yamaguchi, 2017). TBPH is a component of omega speckles, and defects in their maturation alter TBPH sub-cellular localisation (Lo Piccolo et al., 2018). Moreover, expression of the human TDP-43 in fly heads increases hsrω levels, while loss of one copy of *hsrω* mitigates TDP-43-induced eye degeneration (Chung et al., 2018). Reduced *hsrω* expression also decreases Caz levels and promotes its cytoplasmic localisation (Lo Piccolo and Yamaguchi, 2017). In addition, *hsrω* downregulation alters the methylation status of ectopically expressed FUS via upregulation of *PRMT5*, thereby enhancing its proteasomal degradation (Lo Piccolo et al., 2019). Notably, the human functional homologue of hsrω, Satellite III (SatIII), is upregulated in the frontal cortex of FTD patients with TDP-43 pathology, as well as in human cells overexpressing *TDP-43* (Chung et al., 2018). Finally, knockdown of either *caz* or *hsrω* results in similar motor neurons degeneration and locomotion defects, further supporting a role for lncRNA-mediated regulation in ALS-FTD pathology (Lo Piccolo and Yamaguchi, 2017).

Hexanucleotide repeat expansions in the *C9orf72* gene represent another common cause of ALS/FTD (Abramzon et al., 2020). These expansions contribute to disease pathogenesis through multiple mechanisms, including loss of C9ORF72 protein function, toxic gain of function from repeat RNA, and the production of dipeptide repeat proteins via repeat-associated non-ATG (RAN) translation (Balendra and Isaacs, 2018) (Figure 4). In *C9orf72*-linked ALS/FTD, GGGGCC repeat-mediated toxicity can be mitigated by overexpression of specific RBPs, such as *HNRNPA2B1*, which binds the repeat RNA and reduces its availability (Taminato et al., 2023). Consistently, knockdown of the endogenous homologue *hrb87F* in *Drosophila* models leads to increased levels of repeat RNA, supporting its protective role against GGGGCC repeat toxicity *in vivo* (Taminato et al., 2023) (Figure 4; Table 2). Additionally, *Drosophila* Glo and its human homologue hnRNP F are sequestered into the GGGGCC repeat RNA foci in both fly models and patient samples (Moens et al., 2018; Conlon et al., 2016; Haeusler et al., 2014) (Figure 4).

Overall, evidence from *Drosophila* and human studies highlights a conserved and dynamic hnRNP regulatory network in the ALS/FTD spectrum (Figure 4; Table 2). Interactions between hnRNPs and key disease-associated factors, such as TDP-43, FUS and *C9orf72* repeat expansions, can ultimately modulate, either attenuating or exacerbating, the disease progression (Table 2).

#### 2.2.2 Fragile X-associated tremor/ataxia syndrome (FXTAS)

FXTAS is a late-onset neurodegenerative disorder characterised by the expansion of a 55–200 CGG repeat tract in the 5′-UTR of the *fragile X mental retardation 1* (*FMR1*) gene on the X chromosome, which leads to elevated levels of FMR1 mRNA. Further expansion of these repeats results in a significant reduction or complete loss of the coding protein, FMRP, giving rise to a distinct disorder known as fragile X syndrome (FXS) (Leehey, 2009).

Studies in a *Drosophila* model of human FXTAS have highlighted a rescuing effect activity of the human protein hnRNP A2/B1 and its fly homologues Hrb87F and Hrb98DE (Table 2). Furthermore, hnRNP A2/B1 has been shown to physically associate with CGG repeat RNA in mouse cerebellar lysates, with this interaction being more prominent in the cytoplasm than in the nucleus (Sofola et al., 2007) (Figure 4). Collectively, these findings support the hypothesis that the CGG repeat sequester hnRNP A2/B1 and related proteins, thereby impairing their normal functions and contributing to disease pathology (Sofola et al., 2007). Consistent with this, hnRNP A2/B1, along with several other proteins, has been identified within intranuclear inclusions associated with FMR1 mRNA in FXTAS patients (Iwahashi et al., 2006). Interestingly, when TDP-43 is expressed in an FXTAS *Drosophila* model, it does not colocalise with the CGG repeat but it is still able to mitigate toxicity in collaboration with Hrb87F and Hrb98DE (He et al., 2014). As knockdown of *TBPH* does not exacerbate the CGG repeat toxicity (He et al., 2014), the effect of TDP-43 is thought to reflect a protective gain of function rather than compensation for loss of endogenous activity. Indeed, TDP-43 can correct a mis-splicing event mediated by Hrb87F and Hrb98DE that is disrupted in FXTAS fly models (He et al., 2014) (Figure 4).

Interestingly, in these models, CGG repeat activates retrotransposons, such as *gypsy*, whose activity is regulated by hnRNP A2/B1 in concert with HP1, a key factor in heterochromatin formation and retrotransposon silencing (Klattenhoff et al., 2009; Tan et al., 2012b). Moreover, in *Drosophila* cells, HP1 associates with both Hrb87F and Hrb98DE, and this interaction has also been documented *in vivo* for Hrb87F in the context of heterochromatin organisation (Tan et al., 2012b; Piacentini et al., 2009).

*Drosophila* expressing CGG repeat also displays an altered miRNA expression profile. Notably, the overexpression of *miR-277* enhances the degradation of its targets (*e.g., Drep-2* and *Vimar*), which are thought to contribute to the increased cell death observed in FXTAS models (Tan et al., 2012a). Importantly, the expression of *miR-277* is regulated by hnRNP A2/B1, suggesting that its dysregulation may results from the sequestration of hnRNP A2/B1 at the CGG RNA repeat loci (Tan et al., 2012a).

Altogether, findings from *Drosophila* FXTAS models and human studies point to an additional conserved mechanism underling the CGG repeat RNA toxicity (Figure 4), while further highlighting the value of fly models for identifying protective modifiers such as TDP-43 and hnRNP A2/B1 (Table 2).

#### 2.2.3 Emerging roles of *Drosophila* hnRNPs in neurodegenerative diseases: current evidence and future opportunities

Although the contribution of hnRNPs to a wide range of neurodegenerative diseases has been extensively characterised in humans (Low et al., 2021; Tilliole et al., 2024; Brandão-Teles et al., 2024; Lourinho et al., 2025), corresponding evidence from *Drosophila* remains comparatively limited. Nevertheless, currently available studies point to potentially conserved roles of *Drosophila* hnRNPs in neuronal dysfunction and disease-relevant contexts.

For example, PTBP1 regulates the alternative splicing of the *microtubule-associated protein tau* (*MAPT*) transcript by repressing exon 10 splicing, thereby controlling the production of 3R *versus* 4R tau isoforms (Wang et al., 2004). This balance is critical, as altered isoform ratios are linked to tauopathies and associated neurodegeneration (Zhang et al., 2022). Consistent with this, the *Drosophila* homologue Heph has been identified as a modifier of human Tau-induced toxicity in a fly model of Alzheimer’s disease (Feuillette et al., 2020; Jeon et al., 2020).

hnRNPs have also been implicated in spinocerebellar ataxia-type 31 (SCA31), a disorder caused by pentanucleotide repeat expansions (*e.g.*, UGGAA) in two genes, *brain expressed associated with NEDD4-1* (*BEAN1*) and *thymidine kinase 2* (*TK2*). In *Drosophila* models of SCA31, the ectopic expression of *TDP-43*, *FUS*, or *HNRNPA2B1* independently suppresses the UGGAA-induced toxicity, likely by acting as RNA chaperones that modulate RNA structure and foci formation (Ishiguro et al., 2017). On the other hand, knockdown of the endogenous hnRNPs such as *nonA* or *TBPH* exacerbates the eye degeneration of the model, supporting the thesis in which these proteins are sequestered by toxic RNA repeats (Ishiguro et al., 2017) (Table 2). Intriguingly, expanded UGGAA repeat can also mitigate the toxicity caused by mutant forms of TDP-43, FUS, and hnRNP A2/B1 in flies, suggesting an additional layer of regulatory interplay among hnRNPs (Ishiguro et al., 2017; Ishiguro et al., 2021).

Finally, *Drosophila* studies indicate a conserved link between hnRNPs, RNA metabolism, and mitochondrial dysfunction in Parkinson’s disease-related pathways. The fly protein Glo and its human homologue hnRNP F repress the translation of nuclear-encoded mitochondrial respiratory chain transcripts (Gehrke et al., 2015). Their activity is modulated by PINK1 and Parkin, which promote monoubiquitination of Glo/hnRNP F, likely altering their RNA-binding properties and releasing target mRNAs for translation. In this way hnRNPs provide a mechanistic bridge between RNA regulation and mitochondrial homeostasis, with implications for disease progression (Gehrke et al., 2015).

Overall, although still underexplored in *Drosophila*, current evidence supports a broader and evolutionarily conserved role of hnRNPs in neurodegenerative disease, spanning tauopathies, repeat expansion disorders, and Parkinson’s disease.

## 5 Conclusion

In this review, we examined the roles of heterogeneous nuclear ribonucleoproteins (hnRNPs) in physiology and neurodegenerative disease across *Drosophila* and humans. These evolutionarily conserved regulators integrate multiple layers of RNA metabolism, including alternative splicing control, RNA granule dynamics, RNA transport and translation, processes that are tightly linked to development and disease progression, with profound effect on the nervous system. Across both species, hnRNPs reveal a strong often conserved function in neurodevelopment and neuronal physiology, underscoring the value of *Drosophila* as a model system to dissect fundamental regulatory networks. At the same time, *Drosophila* has proven highly effective for studying neurodegenerative mechanisms, particularly those involving TDP-43 and FUS. However, significant gaps remain, as many hnRNPs that are well characterised in human disease contexts have been comparatively underexplored in flies. Expanding the genetic and functional analysis of hnRNPs in *Drosophila* therefore represents a powerful approach to identify conserved regulatory nodes and disease modifiers. Such effort will contribute to a more integrated and mechanistic understanding of hnRNPs function and dysfunction in neurological disorders.
